# A265 TEMPORAL DYNAMICS OF SYMPTOMS AND GUT MICROBIOTA IN EPISODES OF IRRITABLE BOWEL SYNDROME

**DOI:** 10.1093/jcag/gwac036.265

**Published:** 2023-03-07

**Authors:** V Mohan, M Pinto-Sanchez, A Nardelli, M Magee, R Borojevic, G De Palma, P Britz-McKibbin, S Collins, P Bercik

**Affiliations:** 1 Department of Medicine; 2 Department of Chemistry and Chemical Biology, McMaster University, Hamilton, Canada

## Abstract

**Background:**

Irritable bowel syndrome (IBS) is a disorder of gut-brain axis that manifests with chronic abdominal pain, altered bowel habits, and frequent psychiatric comorbidities. Despite mounting evidence showing gut microbiota composition and associated metabolites being altered in IBS, the mechanisms by which they drive the symptoms are unclear. We have previously shown that several IBS symptoms co-occur, that their severity vary among IBS subtypes (10.1093/jcag/gwab049.050), and that several bacterial taxa are differentially modulated during periods of symptom flares and remission. Here we investigate whether the changes in gut microbiome and bacterial metabolites are linked to symptom occurrence and severity.

**Purpose:**

To investigate temporal associations of IBS symptoms with gut microbiota profiles and metabolites.

**Method:**

16S rRNA gene sequencing was performed on stool samples of 28 IBS patients (IBS-D n=20, IBS-C n=8) and 10 healthy controls (HC), collected weekly over a period of 25 weeks, during which gut and mood symptoms were recorded (total of 950 samples). Correlations between principal ordinates obtained from symptom scores and microbiota beta diversity were studied using Procrustes analysis in R. Metabolomics was performed by Mass Spectrometry and analysed using MetaboAnalyst 5.0. Statistical significance was set at p<0.05.

**Result(s):**

Significant correlation was found between the symptom scores and microbiota beta diversity ordinates over time in 7 patients (5 IBS-D and 2 IBS-C subjects) out of 28 IBS patients. Metabolomics performed on samples selected based on Procrustes analysis and symptom severity scores of individual subjects showed that several pathways are altered in IBS patients (both subtypes) compared to HC, including primary bile acid biosynthesis, beta alanine metabolism, pyrimidine and histidine metabolism. Furthermore, during symptom flares, ornithine, citrulline and gluconic acid vary in IBS-D, while amino acids cysteine, methionine, threonine, and glycine vary in IBS-C subjects.

**Conclusion(s):**

Our results suggest that IBS symptoms and changes in gut microbiota composition and metabolites must be studied in conjunction, in order to understand the mechanisms underlying IBS pathophysiology. We found that same pathways are altered in IBS subjects irrespective of their subtype, suggestive of a basal metabolic shift in IBS patients. This shift may make them sensitive to further gut microbial modulation of carbohydrate and protein metabolism in IBS-D and IBS-C subjects, respectively, leading to symptom flares. Further analyses are needed to investigate these metabolites and associated bacteria, as they can help identifying subsets of patients with specific disease mechanisms. This will be a steppingstone in moving away from symptom-based subtyping towards mechanism-based grouping and developing effective treatment strategies accordingly.

**Disclosure of Interest:**

None Declared

